# Sleep and Cognition in the Elderly

**DOI:** 10.3389/fneur.2013.00071

**Published:** 2013-06-10

**Authors:** Géraldine Rauchs, Julie Carrier, Philippe Peigneux

**Affiliations:** ^1^Unité de Recherche, INSERM-EPHE-Université de Caen Basse-NormandieCyceron, France; ^2^Center for Advanced Research in Sleep Medicine, Hôpital du Sacré-Coeur de MontréalMontréal, QC, Canada; ^3^Neuropsychology and Functional Neuroimaging Unit, Université Libre de BruxellesBruxelles, Belgium

In the past decade, our understanding of sleep mechanisms and their role in cognitive processes including memory functions has markedly increased. However, most data have been gathered in young adults, neglecting the fact that sleep is an age-dependent evolutionary process featuring substantial physiological changes that may impact on daily cognitive functioning. Despite the importance of this topic from scientific and societal standpoints, studies jointly investigating aging, sleep, and cognition remain scarce, even considering patients with neurodegenerative diseases. With this special topic, we aim at providing the reader with an updated overview of those studies assessing the impact of age-related changes in sleep and sleep regulation on various domains of cognition. In this respect, this issue addresses changes in sleep and circadian rhythms in the elderly, and how they impact on cognitive performance and brain activity (Schmidt et al., 2012). Sleep-dependent memory consolidation and the age-related changes that may compromise this complex process are also discussed (Harand et al., 2012), as well as how pre-sleep learning can improve sleep continuity, stability, and organization in older adults (Conte et al., 2012). Considering mental productions during sleep, variations in dream recall frequency, and dream theme diversity across the lifespan are also investigated (Nielsen, 2012). From another perspective, the potential mechanisms underlying sleep changes in adults are investigated, focusing on the role of adenosine in protecting from neurobehavioral impairments after sleep deprivation in older adults (Landolt et al., 2012) and on age-related changes in slow oscillations during sleep-dependent memory consolidation processes (Fogel et al., 2012). Finally, common sleep-related pathologies are addressed. In the context of aging, insomnia complaints in older adults and its neural substrates are a crucial issue (Stoffers et al., 2012), but elderly are also a population at risk for obstructive sleep apnea, which might markedly impact on cognitive processes (Sforza and Roche, 2012). Also, less frequent in isolation in normal aging but commonly associated with dementia with Lewy bodies or Parkinson's disease, REM sleep behavior disorder may accelerate cognitive decline (Gagnon et al., 2012).

Altogether, the contributions in this issue show that a better understanding of age-related changes in sleep architecture and microstructure, of their potential impact on cognition and of their underlying mechanisms is essential to develop efficient care of sleep disturbances in the elderly. Such information is even more crucially needed to better apprehend and treat sleep disturbances in neurodegenerative diseases, such as Alzheimer's disease, where sleep disturbances, taken as downstream symptoms of the disease, can be evidenced years before the diagnosis. These sleep disturbances may significantly accelerate cognitive decline (e.g., Rauchs et al., 2008; Hot et al., 2011; Westerberg et al., 2012) and exacerbate the neuropathological processes leading to amyloid depositions (Kang et al., 2009; Ju et al., 2013). Hence it highlights the utmost importance of preserving sleep quality in older adults for optimal cognitive functioning and opposing to the course of neurodegenerative diseases.
